# Fibroblast growth factor 11 (FGF11) promotes non-small cell lung cancer (NSCLC) progression by regulating hypoxia signaling pathway

**DOI:** 10.1186/s12967-021-03018-7

**Published:** 2021-08-17

**Authors:** Xiaowei Wu, Minjie Li, Ying Li, Yu Deng, Shun Ke, Fan Li, Yujin Wang, Shuchang Zhou

**Affiliations:** 1 Department of Thoracic Surgery, Ersity of Science and Technology, Tongji Hospital, Tongji Medical Collage of Huazhong Univ, 430030, Wuhan, Hubei China; 2 Department of Thoracic Surgery, Zhongshan Hospital, Xiamen University, Xiamen, 361004 Fujian China; 3 Department of Nuclear Medicine, Dongzhimen Hospital of Beijing University of Chinese Medicine, Beijing, China; 4 Department of Emergency Medicine, Tongji Hospital, Tongji Medical College of Huazhong University of Science and Technology, 430030, Wuhan, Hubei China; 5 Department of Radiology, Tongji Hospital, Tongji Medical Collage of Huazhong University of Science and Technology, 430030, Wuhan, Hubei China

**Keywords:** NSCLC, FGF11, HIF-1α, Hypoxia, Cell proliferation

## Abstract

**Background:**

Accumulating evidence highlights the critical roles of fibroblast growth factors (FGFs) in regulating the progression of multiple human cancers, including non-small cell lung cancer (NSCLC). In this study, we investigated the role of FGF11 in the progression of NSCLC.

**Methods:**

Previously published transcriptomic data (GSE75037 and GSE81089) were used to compare FGF11 expression level between NSCLC tumor tissues and adjacent normal tissues. 100 cases of NSCLC tumor tissues and 30 cases of matched adjacent normal tissues were used to validate FGF11 expression at mRNA and protein level by qPCR and immunohistochemistry. Bioinformatics analysis and dual luciferase reporter analysis were performed to confirm the regulatory effect of miR-525-5p on FGF11 expression. CCK-8 assay and transwell migration assay were employed to examine cellular proliferation, migration and invasion. Gene set enrichment analysis (GSEA) was performed to identify the signaling pathway associated with FGF11 expression. Finally, the functional role of FGF11 in NSCLC tumor growth was evaluated by in vivo study.

**Results:**

FGF11 was upregulated in NSCLC tumor tissues and tumor cell lines. High FGF11 expression was associated with a poor prognosis in NSCLC patients. In vitro loss- and gain-of function experiments demonstrated that FGF11 knockdown inhibited, whereas FGF11 overexpression promoted the proliferation, migration and invasion of NSCLC cells. Dual luciferase reporter assay confirmed that FGF11 was downregulated by miR-525-5p, and the effect of FGF11 on cell proliferation, migration and invasion could be interfered by miR-525-5p. GSEA analysis further revealed that FGF11 expression was enriched with genes in hypoxia signaling pathway and the oncogenic function of FGF11 could be suppressed by knocking down HIF-1α in NSCLC cells. Moreover, FGF11 knockdown suppressed NSCLC tumor growth whereas FGF11 overexpression promoted tumor growth in vivo.

**Conclusions:**

Our study showed that FGF11 functions as an oncogene in tumor NSCLC progression. miR-525-5p seems to negatively regulate FGF11 and the oncogenic role of FGF11 is dependent on the upregulation of HIF-1α. Our study suggests that targeting FGF11 and HIF-1α may serve as novel strategies for the treatment of NSCLC.

**Supplementary Information:**

The online version contains supplementary material available at 10.1186/s12967-021-03018-7.

## Background

According to the Global Cancer Observatory (GLOBOCAN), the world is facing an increasing incidence and mortality of cancer, and lung cancer is the most prevalent type of cancer causing the mortality [1]. Lung cancer is usually subtyped into small cell lung cancer (SCLC) and non-small cell lung cancer (NSCLC), which account for approximately 20% and 80% of diagnosed cases of lung cancer respectively [2, 3]. Despite the advancement of diagnosis and treatment, the management of NSCLC remains one of the most serious medical challenges [4, 5]. Understanding the factors and mechanisms underlying the progression can provide insights into the development of novel therapeutic strategies targeting NSCLC.

Fibroblast growth factor (FGF) family members play important roles in a variety of tumorigenesis by activating multiple signaling pathways to support cell proliferation and promote epithelial-mesenchymal transition (EMT) [6–8]. Previous report has revealed that downregulating fibroblast growth factor 5 can inhibit the proliferation and invasion of NSCLC cells [9]. As one important member of FGF family, the implication of FGF11 and the relevant signaling processes remain to be investigated in NSCLC [10, 11].

microRNAs (miRNAs) are small non-coding RNAs which function in regulating mRNA transcription and post-transcriptional stability [12, 13]. The dysregulation of miRNAs has been implicated in a wide spectrum of human diseases especially in the progression of different types of cancers, including gastric cancer [14], breast cancer [15], lung cancer [16] and colorectal cancer [17].

At present, the prognostic role of FGF11 was only reported in rare cancers, such as nasopharyngeal carcinoma [18] and prostate cancer [19]. Its roles in other physiological processes such as bone resorption and endothelial tube formation have also been reported [20, 21]. A previous study showed that copy number variations (CNVs) of FGF11 gene was correlated with the risk of lung cancer in heavy smokers [22], suggesting a potential role of FGF11 in lung cancer initiation. Hypoxia induced factors-1 alpha (HIF-1α) plays a key role in hypoxia signaling pathways which is dysregulated lung cancer [23–26]. FGF11 could be induced by hypoxia, which in turn promotes the function of HIF-1α in a positive feedback loop in endothelial cells [21]. However, the functional role of FGF11-HIF-1α axis in cancer development remains unclear.

Through the analysis of previously published transcriptomic data, we found that FGF11 was upregulated in NSCLC tumor tissues, which was also associated with poor prognosis of the patients. Based on target prediction using multiple miRNA databases, we found that miR-525-5p could target and suppress FGF11 expression, suggesting that miR-525-5p is a negative regulator of FGF11. GSEA analysis further revealed that in lung tumor tissues, a high expression of FGF11 was enriched with genes in hypoxia signaling pathway. Functional experiment provided evidence that the oncogenic function of FGF11 could be suppressed by knocking down HIF-1α in NSCLC cells. In summary, our study indicates that FGF11 is an oncogenic factor in NSCLC. We identified miR-525-5p and HIF-1α as the negative and positive regulator in FGF11-dependent function, suggesting that targeting these molecules can serve as novel therapeutic approaches for treating NSCLC.

## Material and methods

### Patients and clinical follow up

100 cases of NSCLC tumor tissues and 30 cases of matched adjacent normal lung tissues were obtained from patients who were newly diagnosed with NSCLC by histopathological analysis. The samples were collected by surgery at Tongji Hospital. All the patients did not receive any other treatment before surgical operation. After surgery, all the samples were snap-frozen in liquid nitrogen. The use of human samples was approved by the Ethics Committee of Tongji Hospital, and all patients and/or their relatives have signed the informed consents. To monitor the survival condition, all the patients were clinically followed for four years. Patients who failed to return for clinical observation or died of accidents were excluded from survival analysis. The clinical characteristics of the 100 NSCLC patients (Histology, Stage, etc.) were shown in Additional file 1: Table S1. Patients were classified into FGF11 high and FGF11 low group (n = 50 in each group) based on the median of FGF11 level quantified by qPCR analysis in their NSCLC samples. The median expression level is 6.40 (FGF11 normalized to GAPDH expression).

### Cell culture

Four human NSCLC cell lines (A549, NCI-H460, CALU3, H1975), and human normal lung epithelial cell line (BEAS-2B) were purchased from ATCC (Manassas, VA, USA). All cells were cultured in RPMI 1640 medium (Gibco, USA) which supplemented with 10% FBS (Gibco, USA), 100 U/ml penicillin and 100 mg/ml streptomycin at 37 °C in a humidified incubator with 5% CO_2_.

### RNA extraction and qPCR analysis

Total RNA was purified from NSCLC tumor cell lines (~ 5 × 10^^5^ cells) and tissues (1 ~ 2 g) using RNeasy mini kit (Qiagen, USA). 5 µg purified total RNA was reverse transcribed into cDNA using the PrimeScript RT reagent kit (Cat# RR037A, Takara, China). miRNA was transcribed using the miRNA specific RT primers which were designed and synthesized by Guangzhou RiboBio, Co., Ltd.

Quantitative real-time PCR (qPCR) reactions were performed on ABI Prism 7500 real time PCR instrument (Applied Biosystems, CA, USA) using the SYBR Green PCR Master Mix (Takara). The relative expression of target gene was determined by 2^−ΔΔCt^ method. GAPDH and U6 was selected as the internal control genes for normalizing mRNA and miRNA expression respectively. Primer sequences of FGF11, HIF-1α and GAPDH, U6 and miR-525-5p were listed as following: FGF11 forward: 5′-GGCATCGTCACCAAACTGTT-3′; reverse: 5′-GCAGTCCCTCAGCATTCATG-3′; HIF-1α forward: 5′-GACAGCCTCACCAAACAGAG-3′; reverse: 5′-GTAGCTGCATGATCGTCTGG -3′; GAPDH forward: 5′-CTGACTTCAACAGCGACACC-3′; reverse: 5′-CTGACTTCAACAGCGACACC-3′; U6 forward: 5′-GCTTCGAGGCAGGTTACATG-3′; reverse: 5′-GCAACACACAACATCTCCCA-3′. miR-525-5p forward: 5′-GCGGTCCCTCTCCAAATGT-3′ reverse: 5′-AGTGCAGGGTCCGAGGTATT-3′.

### shRNA knockdown, siRNA knockdown and FGF11 overexpression

pLKO.1-Puro lentiviral vector was used for shRNA-mediated gene silencing, and pLenti-puro plasmid was used for FGF11 overexpression. Lentiviral plasmids overexpressing FGF11 (containing cDNA of FGF11) or carrying FGF11-shRNA and sh-Negative Control (sh-NC) were constructed by GenePharma Co. Ltd. (Shanghai, China). The packaging of recombinant lentivirus was performed in 293 T cells by GenePharma Co. Ltd. (Shanghai, China). To generate stable shRNA-mediated knockdown or FGF11 overexpression, 1 × 10^5^ cells were seeded in a 24-well plate. When cells reached at 50 ~ 60% confluence, cells were infected with recombinant lentivirus at a MOI (multiplicity of infection) = 5, in the presence of 10 µg polybrene (Sigma, tr-1003-g). Infected cells were selected with 1.0 μg/mL puromycin for two weeks to eliminate the uninfected cells before further experiment. qPCR and western blot were performed to confirm the efficiency of shRNA-mediated knockdown and FGF11 overexpression.

miR-525-5p mimic and miR-NC were purchased from Guangzhou RiboBio (Guangzhou, China): miR-525-5p mimic: 5′-CUCCAGAGGGAUGCACUUUCU-3′.

miR-NC: 5′-UUCUCCGAACGUGUCACGU-3′. Control siRNA and HIF-α siRNA (sc-39464) were purchased from Santa Cruz Biotechnology (Texa, USA). The miRNA mimic/inhibitor and siRNAs were transfected into cells using Lipofectamine 3000 (Invitrogen, L3000001) according to the manufacturer’s instructions. 50 nM of each molecule was used for transfection and functional experiments were performed 48 h post-transfection.

### Dual luciferase reporter assay

To demonstrate the functional interaction between FGF11 and miR-525-5p, FGF11 3′UTR region containing miR-525-5p binding sites (named as WT) and fragment containing site-directed mutagenesis (named as MUT) were cloned into psiCHECK-2.0 vector. The reporter plasmid and Renilla luciferase (hRlucneo) control plasmid were co-transfected into 293 T cells with either MiR-525-5p mimic or inhibitor in a 24-well plate (2 × 10^5 cells/well) using Lipofectamine 3000 reagent according to the manufacturer’s instructions (Invitrogen, L3000001). 48 h post transfection, the relative luciferase activities were measured using Dual-Luciferase Reporter Assay Kit (Promega, E1910) on a luminescence microplate reader (Infinite 200 PRO; Tecan). The relative firefly luciferase activity in the reporter plasmid was normalized to that of Renilla luciferase (hRlucneo) control plasmid.

### Cell Counting Kit 8 (CCK-8) assay

Cell Counting Kit-8 (CCK-8; Dojindo, Japan) assay was used to examine the cell proliferation capacity. NSCLC cells were seeded in a 96-well plate at a density of 3,000 cells/well, then cultured for indicated time period. 10 μl of CCK-8 solution was added to the cell culture at each time point, the plates were incubated for 3 h. 10μL CCK-8 reagent was added to each well at indicated time point and incubated in the incubator for 1 h. A microplate reader (Bio-Rad, CA, USA) was used to detect the absorbance value (OD value) in each well at 450 nm.

### Colony formation assay

Colony formation assay was used to evaluate the long-term proliferation potential. Cells were infected with recombinant lentivirus carrying shRNA or co-transfected with corresponding microRNA mimics, inhibitor or siRNA, as mentioned in the shRNA and siRNA expression Sect. 48 h post-transfection, cells were seeded into a 6-well plate at the density of 200 cells/well after cell counting. The culture medium was changed every two days. After two weeks’ culture, cells were fixed with paraformaldehyde and stained using Giemsa Stain Kit (Abcam, ab150670) according to the manufacturer’s instructions. Finally, the number of colonies formed in each condition was counted using Leica AM6000 microscope.

### Cell migration and invasion assay

Cell migration ability was determined by wound healing assay and cell invasion assay. For wound healing assay, cells were seeded into a 6-well plate of 2 × 10^5^ cells. When the confluency reached at 70–80%, a scratch was created on cell monolayer using a sterile 100 μl tip. Cells were cultured for another 48 h and the closure of the scratch was imaged using a phase contrast microscope (Leica AM6000 microscope.).

For the invasion assay, Matrigel (354,230, BD, USA) was diluted in an appropriate proportion and coated on the bottom of transwell chamber (CLS3398, Sigma, Germany). Cells were trypsinized and then resuspended in serum-free medium. After cell counting, 1 × 10^4^ cells were seeded into the upper chamber in medium with 3% FBS, and 500μL medium with 10% FBS was added into the lower chamber. After incubator for 48 h, cells were fixed with 4% paraformaldehyde for 10 min at room temperature and staining with 0.5% crystal violet solution (c0755, Sigma, Germany) in 25% methanol for 20 min. The invading and migrating cells were image and counted using Leica AM6000 microscope.

### Apoptosis assay by flow cytometry

Apoptosis assay was performed using the PE-Annexin V Apoptosis Detection Kit I (BD biosciences, 559763). After lentiviral infection or transfection with microRNA mimics, cells were washed twice with pre-cold PBS. Cells were harvested by trypsinization and collected by centrifuging at 500xg for 5 min. The cell pellet was resuspended in 1 ml medium with 5 μl of PE-Annexin V and 5 μl of 7-AAD solution for15 min at room temperature. Stained cells were analyzed using Attune NxT Flow Cytometer (BD, Biosciences, USA). Collected data were analyzed by Flow Jo software (Version 13.0, BD).

### Western blot analysis

Total protein was extracted from NSCLC tumor tissues and cells using RIPA lysis buffer containing protease inhibitor cocktail (Invitrogen, USA), then quantified by a BCA Protein assay kit (Solarbio, Beijing, China). 10 ug protein was used for SDS-PAGE electrophoresis. Separated protein in SDS_PAGE gel was transferred onto the PVDF membrane (BioRed, USA). After blocking with 5% skimmed milk for 1 h, the membrane was then incubated with primary antibodies: FGF11 (1:1000; Cell Signaling Technologies #3139, MA, USA), HIF-1α (1:1000, Cell Signaling Technologies #3716), β-Actin (13E5) (1:2000, Cell Signaling Technologies #4970) and GAPDH (1:2000; Cell Signaling Technologies #2118) for 2 h or overnight at 4℃. The membrane was washed 3 times with TBST for 5 min each. After wash, the membrane was further incubated with HRP-linked secondary antibody (1:3000; Cell signaling #7074, MA, USA) at room temperature for 1 h. Then the membrane was washed 4 times with 1 × TBST and the protein bands were visualized using an enhanced chemiluminescence kit (Santa Cruz, TX, USA) and photographed on a gel imager system (Bio-Rad).

### Animal tumor xenograft model

Balb/c nude mice (female, aged 6–8 weeks, weight 18–20 g) were used to perform the in vivo animal experiment. Mice were purchased from the Slac Laboratory Animal Center (Shanghai, China) and maintained under pathogen-free conditions. 5 × 10^5^ A549 and/or NCI-H460 cells stably infected with FGF11, FGF11 shRNA or sh-NC lentivirus were resuspended in 100 μl of PBS and subcutaneously injected into the right flank of mice (5 mice in each group). The tumors were monitored using a caliper weekly and the tumor volume was calculated using the formula: V(tumor) = 0.5 × length × width^2^. Seven weeks after tumor cell inoculation, all the mice were euthanized by CO_2_ asphyxiation, and the xenograft tumors were excised and weighted. All experimental procedures of the use of animal were approved by the Ethics Committee of Tongji Hospital. All the efforts were made to minimize animal suffering according to the NIH Guide for the Care and Use of Laboratory Animals.

### IHC and H&E staining

Immunohistochemical staining (IHC) of Ki-67 protein or FGF11 was performed on 4-mm sections of formalin-fixed paraffin-embedded (FFPE) tumor tissue using VENTANA BenchMark Special Stain platform (Roche, Indianapolis, IN, USA) based on the manufacturer’s instructions. Antibody used for Ki-67 IHC staining was Anti-Ki67 antibody (ab15580, 0.5 µg/ml in TBST, Abcam, USA). Antibody used for FGF11 IHC staining was Anti-FGF11 antibody [MM0282-6J20] (ab89713, 0.5 µg/ml in TBST, Abcam, USA).

Hematoxylin and Eosin (H&E) staining was performed using H&E Stain Kit (ab245880, Abcam, USA). Deparaffinized/hydrated section was incubated in adequate Hematoxylin solution for 5 min. The section was rinsed twice with distilled water to remove excess stain. Then adequate Bluing Reagent was applied to completely cover tissue section and incubate for 30 secs. After washing with distilled water, the section was dehydrated in absolute alcohol, followed by staining with Eosin Y Solution to completely cover tissue for 2–3 min. The section was rinsed using absolute alcohol for three times and mounted in synthetic resin for observation.

### Bioinformatic analysis

The gene expression data of NSCLC patients were retrieved from Gene Expression Omnibus (GEO) repository (GSE75037 and GSE81089). Gene Set Enrichment Analysis (GSEA) was performed according to the instructions on Molecular Signatures Database (MSigDB): https://www.gsea-msigdb.org/gsea/index.jsp. The expression characteristics of FGF11 and the prognosis of NSCLC patients were extracted from TCGA database through the website tool GEPIA (http://gepia.cancer-pku.cn/index.html). MicroRNA target prediction databases were used for predicting FGF11 binding microRNAs: Starbase (http://starbase.sysu.edu.cn/index.php); miRBD (http://mirdb.org/) and Targetscan (http://www.targetscan.org/vert_72/).

### Statistical analysis

All results were analyzed by GraphPad Prism 8.0 software (GraphPad Software, San Diego, CA, USA). Statistical analyses were performed using Student's *t*-test, one-way analysis of variance (0ne-way ANOVA), Kaplan Meier plotter analysis or χ2 test. Correlation between FGF11 and HIF-1α expression in NSCLC tissues was assessed by Pearson’s correlation analysis. *P* < 0.05 was considered as significant statistical difference.

## Results

### FGF11 is upregulated in human NSCLC tissues and significantly correlated with the prognosis

**Fig. 1:**
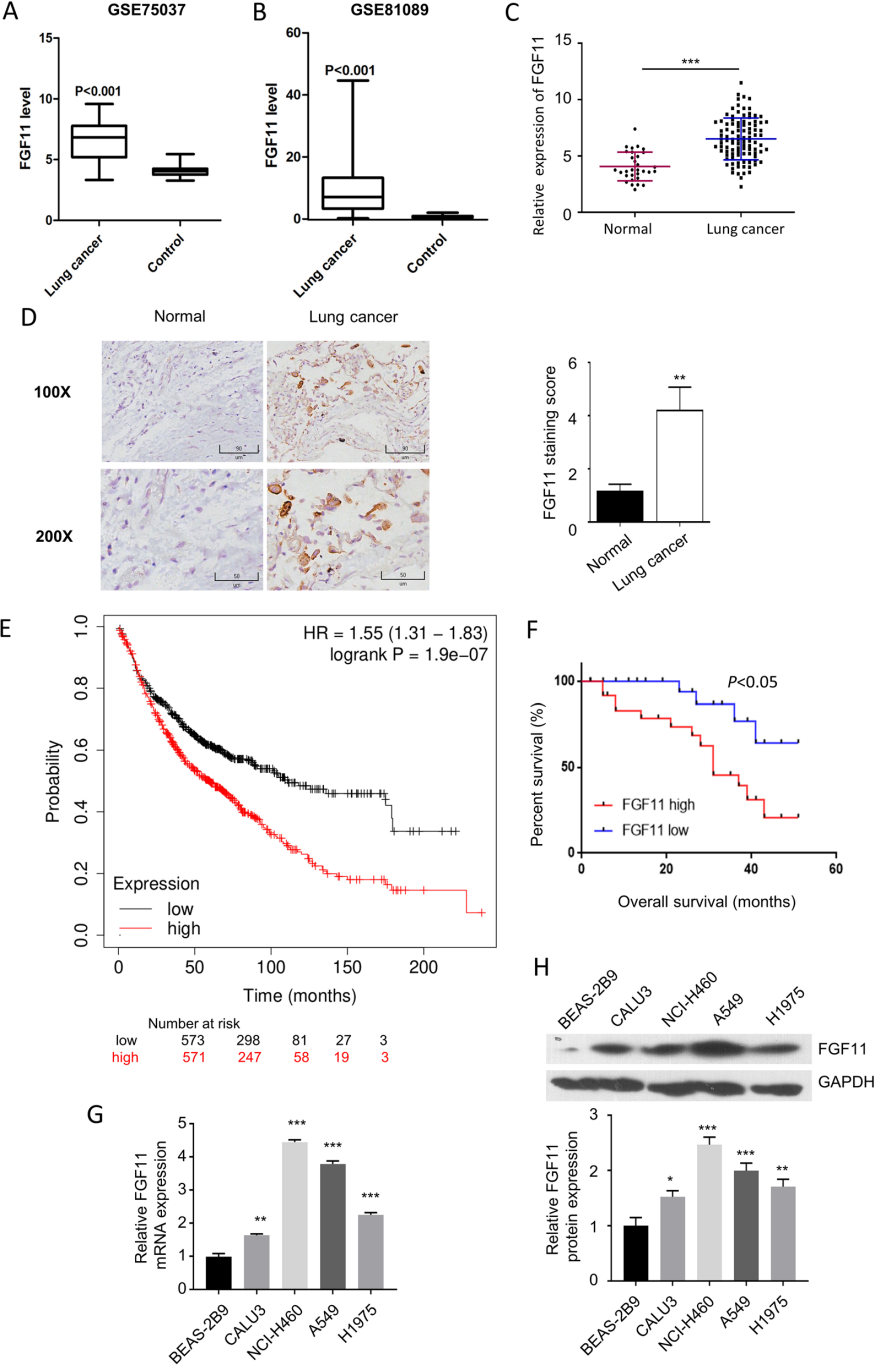
FGF11 is upregulated in human NSCLC tissues and is associated with poor prognosis. **A** The relative expression of FGF11 in GSE75037 dataset. **B** The relative expression of FGF11 in GSE81089 database. **C** qPCR analysis of FGF11 expression in 100 cases of NSCLC tissues and 30 cases of adjacent normal tissues. **D** The examination of FGF11 protein level by immunohistochemistry (IHC) in NSCLC tumor tissue and adjacent normal tissue. **E** Kaplan–Meier plotter survival analysis of 1144 cases of NSCLC patients from TCGA database. **F** Kaplan–Meier plotter analysis of survival probability of FGF11 high expression group (n = 50) and the low expression group (n = 50) in NSCLC patients. **G** qPCR analysis of FGF11 expression in NSCLC cell lines (A549, NCI-H460, CALU3, H1975) and human normal lung epithelial cell line (BEAS-2B). **H** Western bot analysis of FGF11 protein level in NSCLC cell lines (A549, NCI-H460, CALU3, H1975) and human normal lung epithelial cell line (BEAS-2B). The above data in (G and H) are the summary of the measurements of 3 independent experiments (mean ± standard deviation). *P < 0.05, ** *P* < 0.01, ***P < 0.001

We first analyzed the previously published transcriptome data (GSE75037 and GSE81089) containing NSCLC tumor sample and the adjacent normal tissues. We found that the expression level of FGF11 was significantly higher in NSCLC tumor tissues than that of normal tissues (Fig. 1A and B). To further validate this observation, we collected 100 NSCLC tumor tissues and 30 matched adjacent normal lung tissues. qPCR analysis revealed that FGF11 expression level was much higher in NSCLC tissues when compared to the normal tissues (Fig. 1C). Moreover, immunohistochemistry (IHC) analysis confirmed a higher protein level of FGF11 NSCLC tumor tissue (Fig. 1D).

To further evaluate the clinical significance of FGF11 on patient survival, we first selected 1144 cases of NSCLC patients from TCGA database, and allocated them into a FGF11-high expression group (n = 571; > median expression level of all cases) and a FGF11-low expression group (n = 573; < median expression level of all cases). Kaplan Meier plotter analysis showed that high FGF11 level was associated with an overall survival of in NSCLC patients (Fig. 1E). Meanwhile, the survival analysis of NSCLC cases we collected showed a similar tendency (Fig. 1F). We further performed qPCR and western blot analysis using NSCLC cell lines and a human normal lung epithelial cell line (BEAS-2B). mRNA and protein levels were significantly higher in all NSCLC cell lines when compared to normal lung epithelial cell line (Fig. 1G and H). Collectively, those data suggest that FGF11 expression level correlates with the prognosis in NSCLC patients.

### FGF11 knockdown inhibits NSCLC cell proliferation and promotes apoptosis

**Fig. 2:**
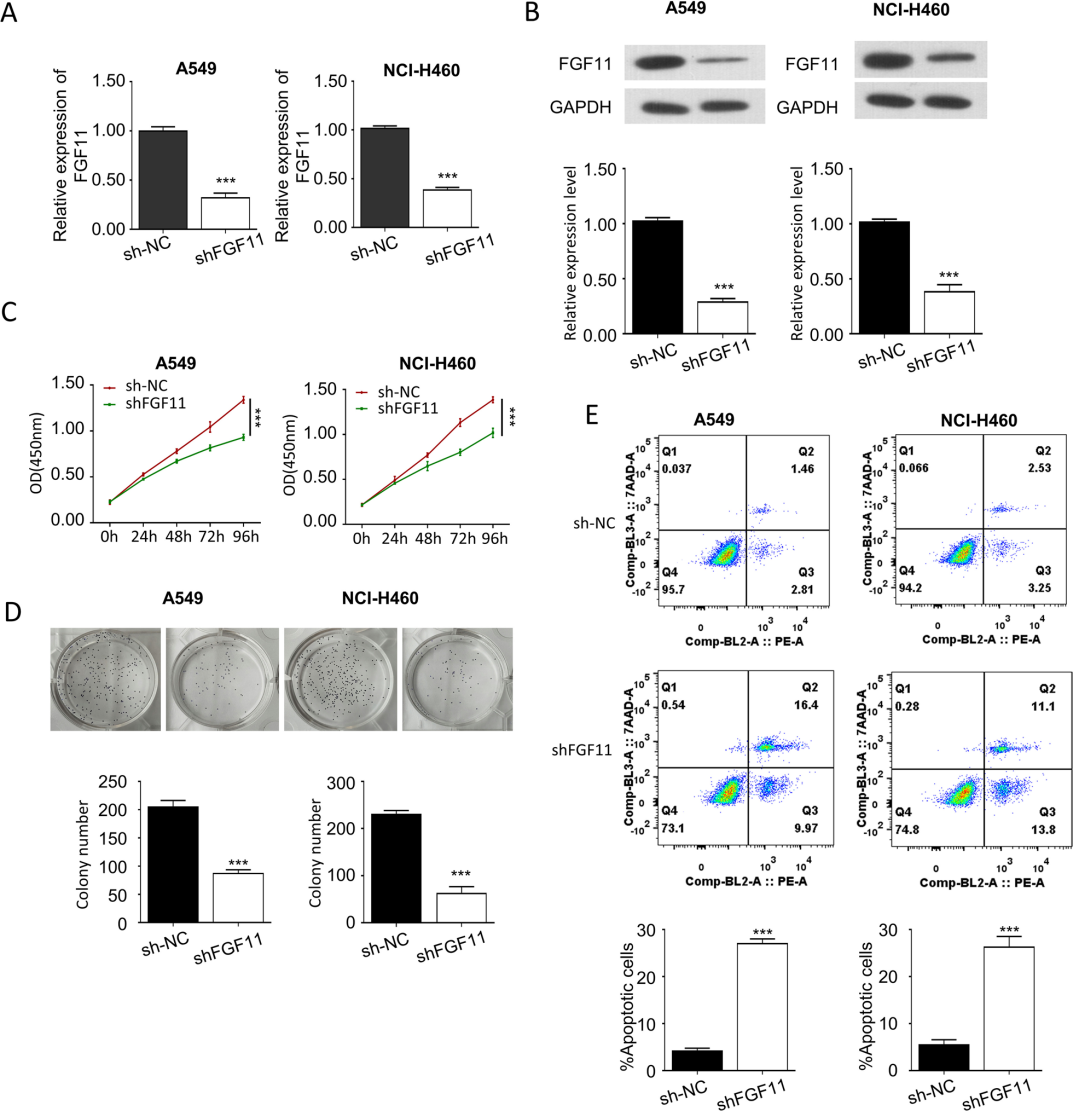
Knockdown of FGF11 inhibits NSCLC cell proliferation and promotes cell apoptosis. Cells were infected with lentivirus carrying FGF11 shRNA (shFGF11) or control shRNA (sh-NC). **A** qPCR analysis of FGF11 mRNA expression in A549 and NCI-H460 cells after shRNA introduction. **B** Western blot analysis of FGF11 protein levels in A549 and NCI-H460 cells. **C** The proliferation of A549 and NCI-H460 cells after shRNA introduction was detected by CCK-8 assay. **D** Colony formation assay of A549 and NCI-H460 cells after shRNA introduction. **E** Flow cytometry analysis of cells stained with Annexin-V and 7-AAD. Data are the summary of the measurements of 3 independent experiments (mean ± standard deviation). *P < 0.05, ** P < 0.01, ***P < 0.005 vs. sh-NC or empty vector-infected cells

The elevated level of FGF11 in human NSCLC samples prompted us to further explore the biological functions of FGF11 in NSCLC cells. FGF11 was silenced in A549 and NCI-H460 cells by infection with FGF11 shRNA (shFGF11) lentivirus. The knockdown efficiency was confirmed by qPCR analysis (Fig. 2A) and western blot (Fig. 2B). CCK-8 proliferation assay was used to examine the cell proliferation capacity after FGF11 silencing in A549 and NCI-H460 cells. The knockdown of FGF11 notably suppressed the cell proliferation in both cell lines (Fig. 2C). The knockdown of FGF11 also significantly inhibited the colony formation ability of NSCLC (Fig. 2D). In addition, apoptosis assay by flow cytometry revealed an increased percentage of apoptotic cells when FGF11 was knocked down (Fig. 2E). The above data demonstrate an indispensable role of FGF11 in supporting NSCLC proliferation.

### FGF11 overexpression promotes NSCLC cell proliferation

**Fig. 3:**
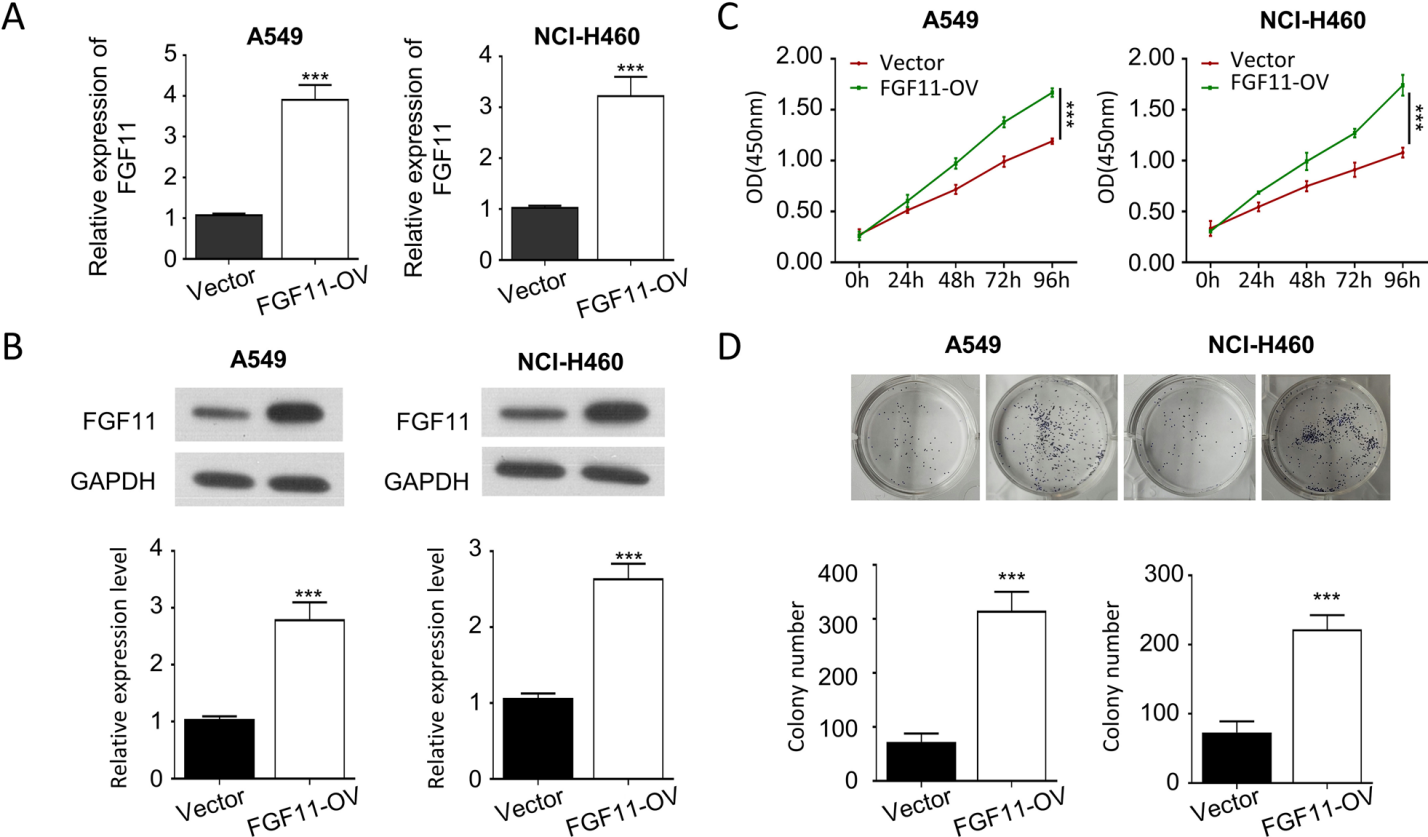
Overexpression of FGF11 promotes NSCLC cell proliferation. Cells were infected with lentivirus carrying FGF11 cDNA clone or empty vector (vector). **A** qPCR analysis of FGF11 mRNA expression in A549 and NCI-H460 cells after lentiviral infection. **B** Western blot analysis of FGF11 protein levels in A549 and NCI-H460 cells. **C** The proliferation of A549 and NCI-H460 cells was detected by CCK-8 assay. **D** Colony formation assay of A549 and NCI-H460 cells after lentiviral infection. Data are the summary of the measurements of 3 independent experiments (mean ± standard deviation). *P < 0.05, ** P < 0.01, ***P < 0.005 vs. NC or empty vector-infected cells

To further support a role of FGF11 in promoting NSCLC cell proliferation, we overexpressed FGF11 in A549 and NCI-H460 cells by infection with lentivirus carrying FGF11 cDNA clone. Lentivirus-mediated overexpression significantly increased the level of FGF11 by about 3 times (Fig. 3A, and B). CCK-8 proliferation assay demonstrated that FGF11 overexpression promoted the tumor cell proliferation as well as the ability in colony formation (Fig. 3C and D), supporting an oncogenic role of FGF11 in NSCLC cells.

### FGF11 promotes NSCLC cell migration in vitro

**Fig. 4:**
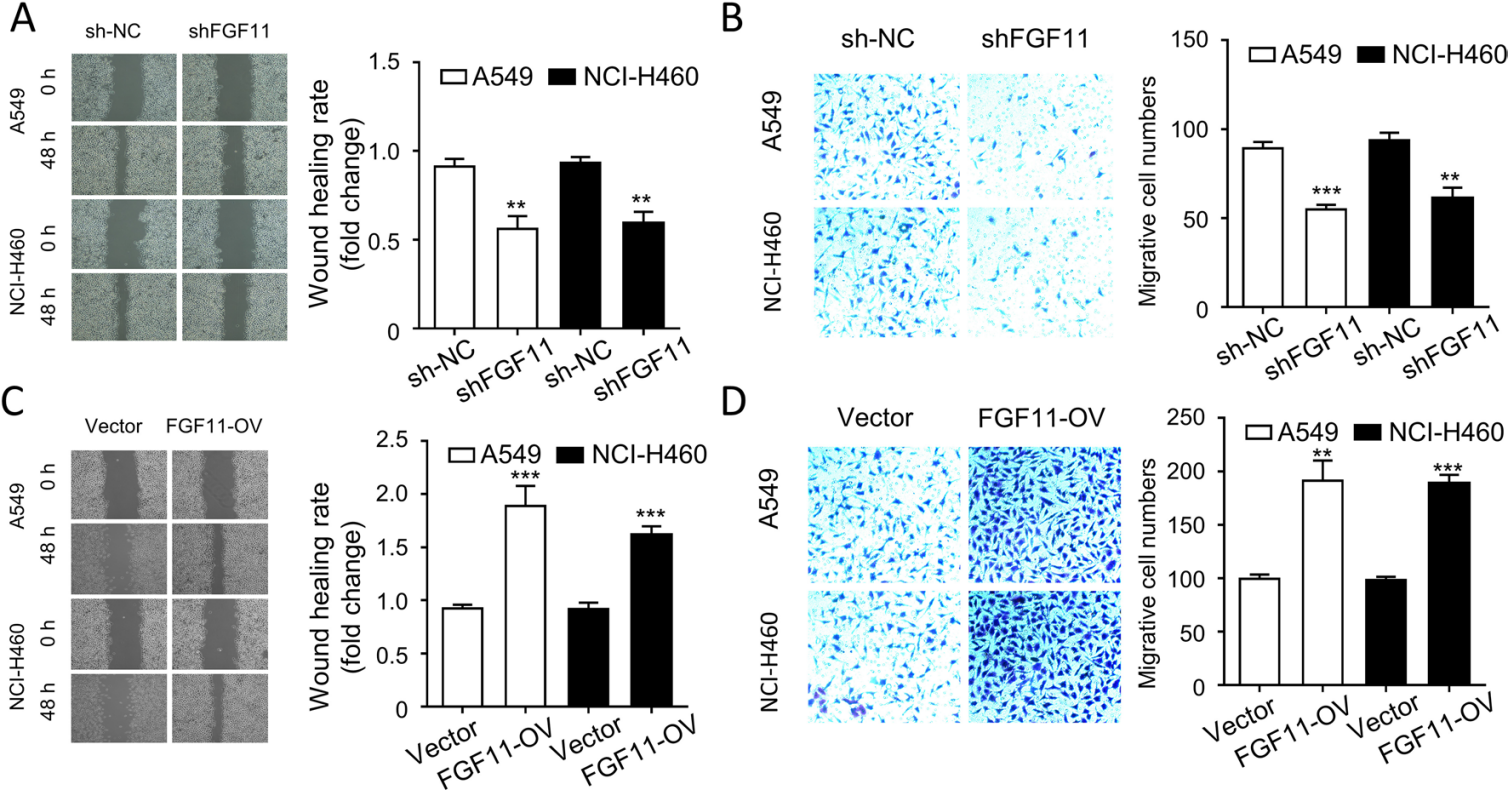
FGF11 promotes NSCLC cell migration in vitro. (A-B) Cells were infected with lentivirus carrying FGF11 shRNA (shFGF11) or control shRNA (sh-NC). **A** Wound healing assay analysis of the migration of A549 and NCI-H460 cells after being infected with FGF11 shRNA. **B** Transwell assay analysis of the migration of A549 and NCI-H460 cells after being infected with FGF11 shRNA. **C**, **D** Cells were infected with lentivirus carrying FGF11 cDNA clone or empty vector (vector). **C** Wound healing assay analysis of the migration of A549 and NCI-H460 cells after FGF11 overexpression. **D** Transwell assay analysis of the migration of A549 and NCI-H460 cells after FGF11 overexpression. The results are represented as mean ± standard derivation. Data are summary of 3 independent experiments. * p < 0.05, ** p < 0.01, *** p < 0.05 *P < 0.05, ** P < 0.01, ***P < 0.005 vs. sh-NC or empty vector-infected cells

We next aimed to evaluate the effect of FGF11 silencing and overexpression on the migration of NSCLC cells. Wound healing assay and transwell assay were performed to assess cell migration ability. We found that the migratory capacities of A549 and NCI-H460 cells were significantly impaired by silencing FGF11 in wound healing assay (Fig. 4A), and transwell assay (Fig. 4B). In contrast, overexpression of FGF11 remarkably enhanced the cell migration in A549 and NCI-H460 cells (Fig. 4C and D). Therefore, the migratory capacity of NSCLC is inversely correlated with FGF11 level.

### FGF11 is negatively regulated by miR-525-5p in NSCLC cells

**Fig. 5:**
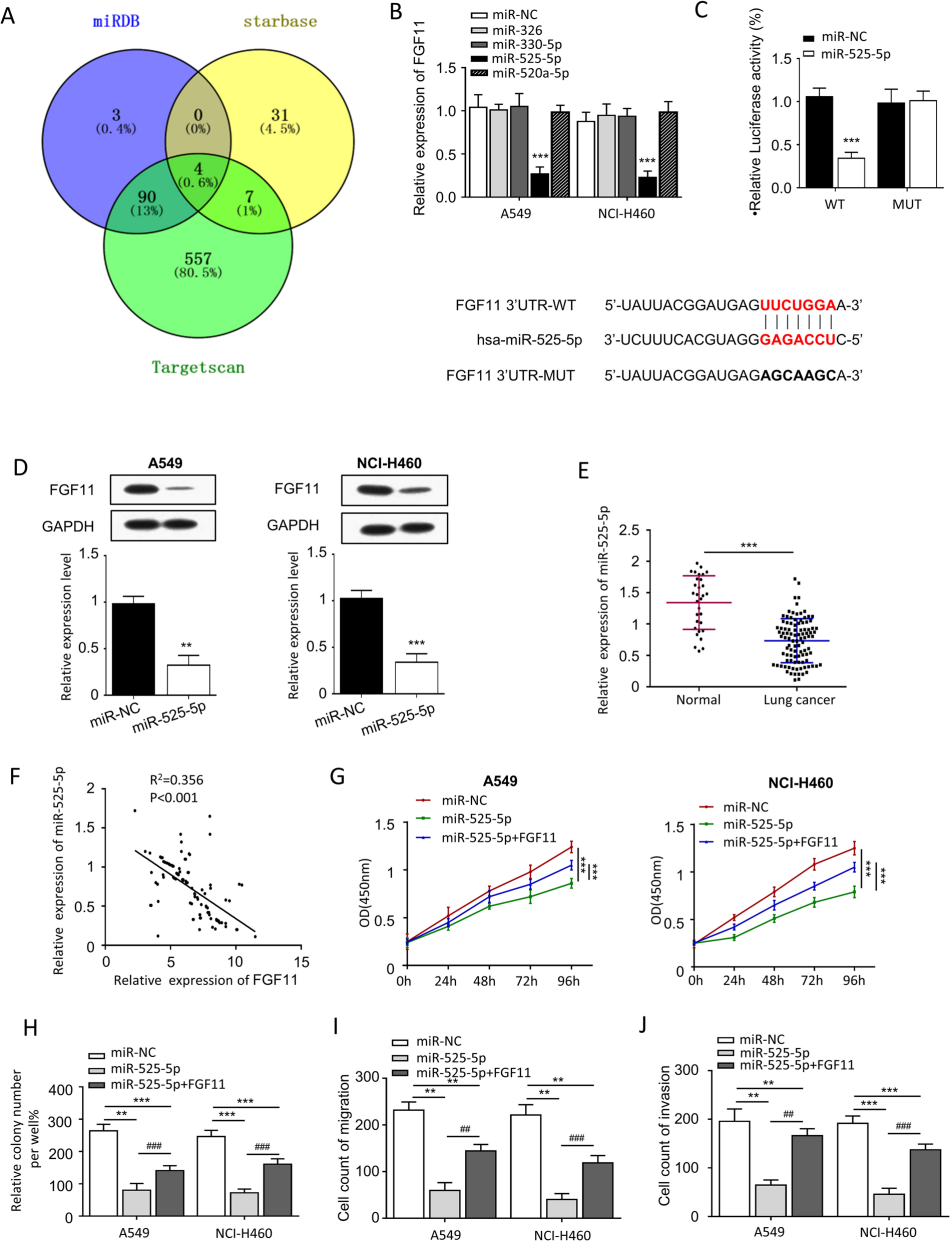
miR-525-5p downregulates FGF11 expression and inhibits NSCLC cell proliferation and migration. **A** Bioinformatic search of miRNAs targeting FGF11 using miRDB, Starbase and Targetscan databases. **B** qPCR analysis of FGF11 mRNA expression in A549 and NCI-H460 cells in the presence of microRNA mimics. **c** Dual-luciferase reporter assay of the effect of miR-525-5p mimic on FGF11 3′UTR. **D** western blot detected the expression of FGF11 after transfected with miR-525-5p. **E** qPCR analysis of the FGF11 mRNA level in NSCLC tumor tissues and normal tissues. **F** The correlation of miR-525-5p level and FGF11 level was examined by Pearson’s correlation analysis. **G** CCK-8 proliferation assay of A549 and NCI-H460 cells transfected with miR-525-5p and/or FGF11. **H** Colony formation assay in A549 and NCI-H460 cells transfected with miR-525-5p and/or FGF11. **I** Wound healing assay in A549 and NCI-H460 cells transfected with miR-525-5p and/or FGF11. **J** Transwell migration assay in A549 and NCI-H460 cells transfected with miR-525-5p and/or FGF11. The results are represented as mean ± standard derivation. Data are summary of 3 independent experiments. *P < 0.05, ** P < 0.01, ***P < 0.005 vs. sh-NC or empty vector-transfected cells

To explore the potential miRNAs regulating FGF11 expression, we first performed target scan of FGF11 sequence using three publicly available miRNA databases (miRDB, Starbase and Targetscan) (Fig. 5A). We found 4 predicted miRNAs (miR-326, miR-330-5p, miR-525-5p, miR-520a-5p) shared by three databases. We introduced miRNA mimics of the 4 microRNAs into A549 and NCI-H460 cells and analyzed FGF11 expression by qPCR. We found that miR-525-5p mimic could significantly reduce the mRNA level of FGF11 (Fig. 5B). We then cloned the wild type binding site in FGF11 3′UTR and the mutated site into a luciferase reporter and performed dual luciferase reporter assay in the presence of control or miR-525-5p mimic. Our results showed that co-transfection of miR-525-5p mimic reduced the relative luciferase activity of WT FGF11 3′UTR but had no detectable effect on the mutated FGF11 3′UTR (Fig. 5C). Meanwhile, western blot analysis also confirmed that miR-525-5p mimic could significantly reduce the protein level of FGF11 in A549 and NCI-H460 cells (Fig. 5D). Furthermore, qPCR was performed to detect the expression of miR-525-5p in NSCLC samples. The result showed that miR-525-5p expression level was significantly lower in NSCLC tumor tissues when compared to normal tissues (Fig. 5E), and there was a negative correlation between miR-525-5p level and FGF11 level (Fig. 5F).

To further examine the function of miR-525-5p on tumor cell proliferation, CCK-8 assay was performed in the presence of absence of miR-525-5p mimic. We found that miR-525-5p mimic could significantly inhibit cell proliferation, whereas overexpression of FGF11 partially rescued cell proliferation in A549 and NCI-H460 cells (Fig. 5G). Colony formation assay also showed the similar result (Fig. 5H). Interestingly, cell migration and invasion ability were also largely suppressed by miR-525-5p mimic (Fig. 5I and J), which could also be rescued by FGF11 overexpression. Together, the above results suggest that miR-525-5p targets FGF11 in the 3′ UTR and negatively control FGF11 expression and NSCLC oncogenesis.

### Hypoxia signaling pathway is involved in FGF11-dependent oncogenic function

**Fig. 6:**
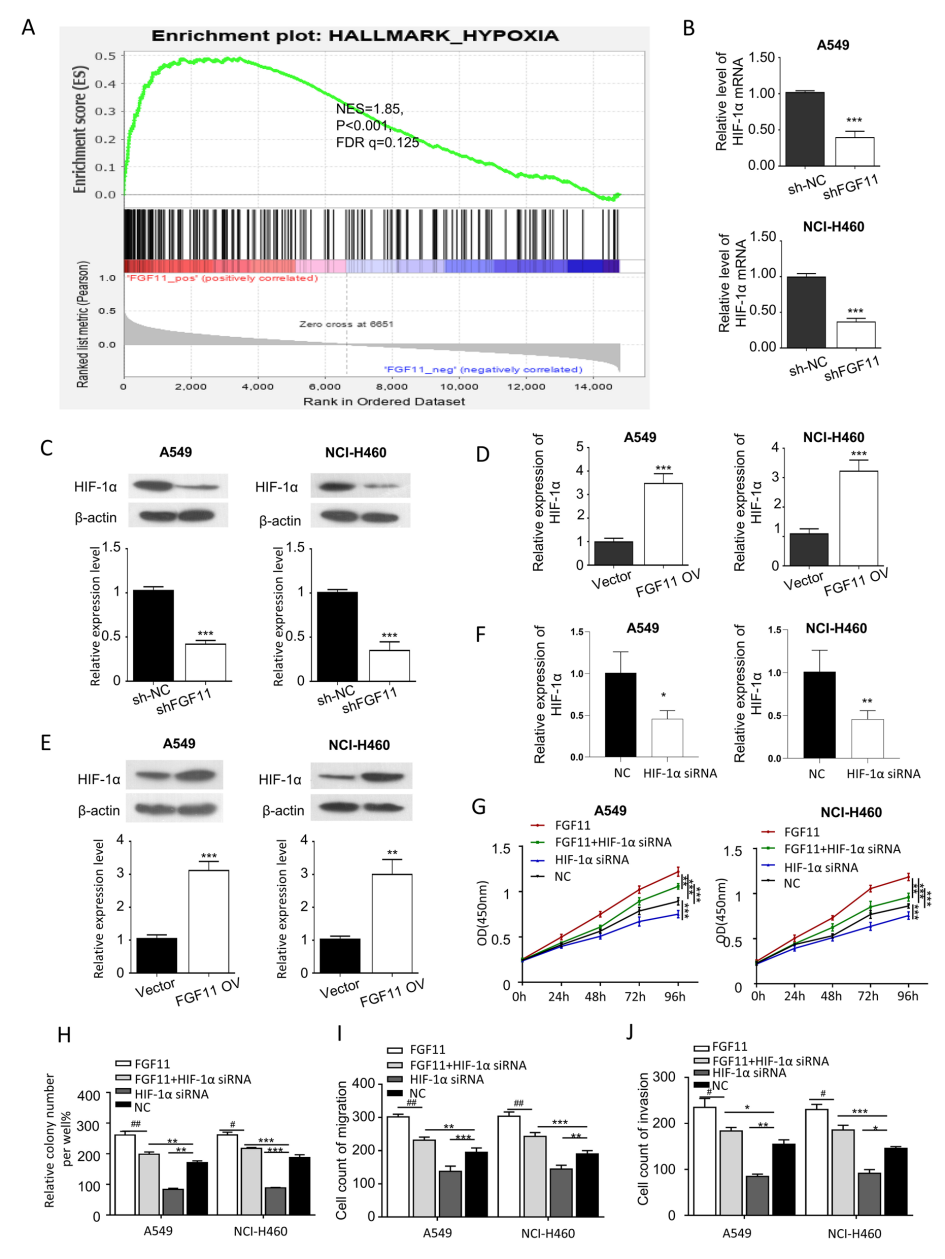
FGF11 regulates HIF-1α expression for NSCLC progression. **A** Gene Set Enrichment Analysis (GSEA) of the hypoxia signaling pathway genes in FGF11 positive and negative samples usingGSE81089 RNA-seq data. **B** qPCR analysis of HIF-1α mRNA expression after FGF11 knockdown in A549 and NCI-H460 cells. **C** Western blot analysis of HIF-1α protein level after FGF11 knockdown in A549 and NCI-H460 cells. **D** qPCR analysis of HIF-1α mRNA expression in A549 and NCI-H460 cells with FGF11 overexpression. **E** Western blot analysis of HIF-1α protein level in A549 and NCI-H460 cells with FGF11 overexpression. **F** qPCR quantification of HIF-1α mRNA level after siRNA transfection. **G** CCK-8 proliferation assay in A549 and NCI-H460 cells transfected with FGF11 and/or HIF-1α siRNA. **H** Colony formation assay in A549 and NCI-H460 cells transfected with FGF11 and/or HIF-1α siRNA. **I** wound healing assay in A549 and NCI-H460 cells transfected with FGF11 and/or HIF-1α siRNA. **J** Transwell migration assay in A549 and NCI-H460 cells transfected with FGF11 and/or HIF-1α siRNA. The results are represented as mean ± standard derivation. Data are summary of 3 independent experiments. *P < 0.05, ** P < 0.01, ***P < 0.005 vs. sh-NC or empty vector-transfected cells

Hypoxia pathway is dysregulated in many cancers, and we next attempted to investigate whether the oncogenic function of FGF11 is related to hypoxia signaling pathway. Gene set enrichment analysis (GSEA) using GSE81089 NSCLC RNA-seq data showed that FGF11 expression was positively enriched with genes in hypoxia signaling pathway (Fig. 6A, NES = 1.85, P < 0.001, FDR q = 0.125 < 0.25). In addition, hypoxia induced factors-1 alpha (HIF-1α) plays a key role in hypoxia signaling pathways which is dysregulated lung cancer [23–26], we next explored whether FGF11 level regulates the expression of HIF-1α in A549 and NCI-H460 cells. Interestingly, the knockdown of FGF11 significantly reduced both the mRNA and protein level of HIF-1α (Fig. 6B and C). In contrast, the relative mRNA and protein level of HIF-1α was increased by FGF11 overexpression in A549 and NCI-H460 cells (Fig. 6D and E).

In addition, to functionally validate the involvement of HIF-1α in cell proliferation, migration and invasion in A549 and NCI-H460 cells, we performed cell proliferation assay and migration assay in the presence of absence of HIF-1α siRNA. qPCR analysis confirmed the downregulation of HIF-1α mRNA after siRNA treatment (Fig. 6F). Our results showed that overexpression of FGF11 could significantly promote A549 and NCI-H460 cells proliferation, but HIF-1α siRNA inhibited the effect of FGF11 overexpression on proliferation (Fig. 6G). Meanwhile, colony formation assay also showed the similar result (Fig. 6H). Overexpression of FGF11 enhanced cell migration in A549 and NCI-H460 cells, and HIF-1α siRNA significantly impaired the effect of FGF11 (Fig. 6I and J). These results indicate that the oncogenic role of FGF11 is dependent on the upregulation of HIF-1α.

### *Knockdown of FGF11 inhibits NSCLC tumor growth *in vivo

**Fig. 7:**
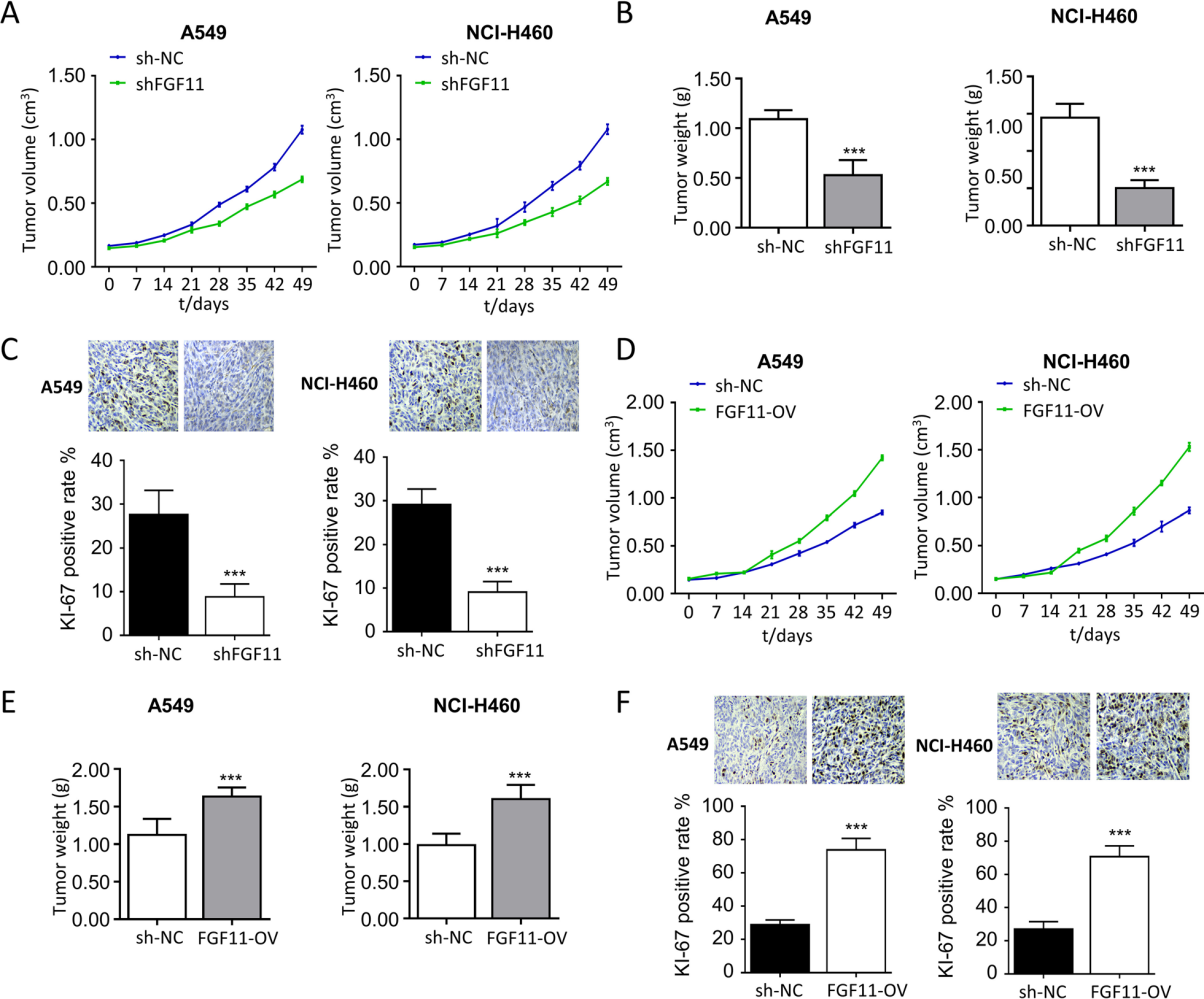
FGF11 knockdown inhibits NSCLC tumorigenesis in vivo. A549 and NCI-H460 cells were infected with FGF11 shRNA and FGF11 overexpression lentivirus. Cells were inoculated into balb/c nude mice (n = 5 mice/group). **A** The tumor growth curve in FGF11 shRNA and control group. **B** Tumor weight quantification in FGF11 shRNA and control group. **C** Immunohistochemistry (IHC) analysis of Ki-67 expression in FGF11 shRNA and control group. **D** The tumor growth curve in FGF11 overexpression and control group. **E** Tumor weight quantification in FGF11 overexpression and control group. **F** Immunohistochemistry (IHC) analysis of Ki-67 expression in FGF11 overexpression and control group. The results are represented as mean ± standard derivation. *P < 0.05, ** P < 0.01, ***P < 0.005 vs. control group

Lastly, we explored the role of FGF11 in NSCLC tumorigenesis using xenograft mouse model. In consistence with the in vitro findings, knockdown of FGF11 resulted in a significantly impaired tumorigenesis of A549 and NCI-H460 cells when injected into the nude mice (Fig. 7A and B). The IHC analysis of Ki67 (cell proliferation marker) in the tumor samples showed that knockdown of FGF11 significantly reduced the percentage of cells expressing Ki-67 (Fig. 7C). In contrast, the overexpression of FGF11 not only promoted the tumor growth rate (Fig. 7D and E), but also increased the percentage of cells expressing Ki-67 in the tumor tissues (Fig. 7F). Collectively, these data support an oncogenic role of FGF11 in tumorigenesis of NSCLC cells.

## Discussion

In recent years, lung cancer has been considered as the most prevalent cancer with highest mortality in Chinese population. Non-small cell lung cancer (NSCLC) accounts for approximately 80% of lung cancer cases, which is characterized as a heterogeneous and multifactorial disease with various genetic mutations. Targeting EGFR (epidermal growth factor receptor) and VEGF (vascular endothelial growth factor) improves the prognosis in NSCLC patients [27–30]. In addition, fibroblast growth factor (FGF) ligands and receptors have been widely implicated in a variety of tumors including lung cancer [31]. For example, FGF5 plays important roles in cell growth and invasion of human NSCLC cells [9]. Analysis of published transcriptomic data revealed that FGF11 was upregulated in NSCLC tissues, but its molecular mechanisms remain elusive.

In this study, we first confirmed the upregulation of FGF11 in both NSCLC tumor tissues and NSCLC cell lines, suggesting an oncogenic effect of FGF11. In vitro gain- and loss-of-function experiments further validated an indispensable role of FGF11 in proliferation, migration and invasion of NSCLC cells. FGF11 also prevents apoptosis in NSCLC cell lines. Besides, the oncogenic activity of FGF11 was verified in the xenograft model using NSCLC cells with stable FGF11 knockdown.

MiR-525-5p has been implicated in multiple cancers, including Glioma [32], Cervical Cancer [33]. The role of miR-525-5p in lung cancer and other human cancers remains to be investigated. Through the microRNA target scan of FGF11 and experimental validation, we found that miR-525-5p negatively regulates FGF11 expression in NSCLC cells, suggesting a tumor-suppressor role of miR-525-5p in the proliferation and migration of NSCLC cells.

Hypoxia signaling pathway has been widely dysregulated in cancer progression, [34, 35]. Previous study highlighted the role of HIF-1α as a key transcriptional regulator in hypoxia signaling pathway [36]. Our results further revealed that FGF11 expression was closely correlated with hypoxia signaling pathway, and validated that FGF11 positively regulated HIF-1α expression. HIF-1α acts as an indispensable signaling transduction mediator by regulating key hypoxia-response genes to support tumorigenesis [37–40]. Consistently, we showed that the knockdown of HIF-1α diminished the oncogenic function of FGF11 in NSCLC cells. We therefore propose that FGF11 might function as an oncogene to upregulate HIF-1α and increase hypoxia signaling pathway to sustain the progression of NSCLC.

In conclusion, our study reveals a novel oncogenic role of FGF11 in NSCLC, which may contribute to the tumorigenesis at least partially through modulating hypoxia signaling pathway. Our findings provide evidence that targeting FGF11 can be a beneficial therapeutic strategy for the treatment of NSCLC patients with elevated FGF11 expression.

## Supplementary Information

**Additional file 1: Table S1.** Clinical pathological features of the selected 100 non-small cell lung cancer patients.

## Abbreviations

FGF: Fibroblast growth factor
NSCLC: Non-small cell lung cancer
HIF: Hypoxia inducible factor
miRNAs: MicroRNAs
CCK-8: Cell counting kit-8
