# Supplementary material for: Fibroblast growth factor 11 (FGF11) promotes non-small cell lung cancer (NSCLC) progression by regulating hypoxia signaling pathway
Source: J Transl Med. 2021 Aug 17;19:353. doi: 10.1186/s12967-021-03018-7 (PMC8369785; doi:10.1186/s12967-021-03018-7)
Supplement: Supplementary file 1 — Additional file 1: Table S1. Clinical pathological features of the selected 100 non-small cell lung cancer patients. [file 12967_2021_3018_MOESM1_ESM.doc]

Additional file 1: Table S1. Clinical pathological features of the selected 100 non-small cell lung cancer patients.

| Clinicopathologic features | Patients (n=100) |
| --- | --- |
| Age median (range) | 65(38-82) |
| Sex, no.(%) |  |
| Male | 64(64) |
| Female | 36(36) |
| Smoking, no.(%) |  |
| Never smoker | 32(32) |
| Ex-smoker | 12(12) |
| Current smoker | 56(56) |
| Histology, no.(%) |  |
| Adenocarcinoma | 58(58) |
| Squamous cell carcinoma | 33(33) |
| Large cell carcinoma | 9(9) |
| TNM clinical stage, no.(%) |  |
| Ⅰ | 71(71) |
| Ⅱ | 14(14) |
| Ⅲ | 11(11) |
| Ⅳ | 4(4) |
| TNM pathological stage. no.(%) | |
| Ⅰ | 55(55) |
| Ⅱ | 13(13) |
| Ⅲ | 30(30) |
| Ⅳ | 2(2) |
| EGFR mutation, no.(%) |  |
| Wildtype | 68(68) |
| Mutant | 32(32) |
| K-ras mutation, no.(%) |  |
| Wildtype | 95(95) |
| Mutant | 5(5) |
